# Role of Fine Needle Aspiration in the Diagnosis of the Rare Disease of Langerhans Cell Histiocytosis in a Child

**DOI:** 10.1155/2014/724895

**Published:** 2014-01-22

**Authors:** Seema Lale, Daniel Soto, Patricia G. Wasserman

**Affiliations:** ^1^Mercy Hospital, 800 Myrtle Street, Independence, KS 67301, USA; ^2^Long Island Jewish Health System, 6 Ohio Drive, Lake Success, NY 11040, USA; ^3^Cytopathology, Columbia University Medical Center, 630 West 168th Street, New York, NY 10032, USA

## Abstract

Langerhan's cell histiocytosis (LCH) results from the proliferation of immunophenotypically and functionally immature, morphologically rounded Langerhan's cells along with eosinophils, macrophages, lymphocytes, and, commonly, multinucleated giant cells. Here we report a case in a 6-year-old boy of differential diagnoses including dermatopathic lymphadenitis (DL), parasitic infection, Kimura's disease, hypersensitivity reactions, cat-scratch disease, sinus histiocytosis with massive lymphadenopathy (SHML), hyperplasic lymph nodes, and lymphoma.

## 1. Introduction

Controversy exists regarding whether the immunologic abnormalities observed in LCH are the cause of the clonal proliferation lesional Langerhan's cells [1]. The etiology of LCH is unknown. Efforts to define a viral cause have not been successful [2, 3]. 

The incidence of LCH has been estimated to be 2 to 10 cases per million children aged 15 years or younger [4, 5]. The male to female ratio is close to one and the median age of presentation is 30 months.

The nomenclature used for LCH indicates the disease extent (i.e., single organ, single system, multisystem, or diffuse). Prognosis and treatment are closely linked to the extent of disease at presentation and whether high-risk organs (spleen, liver, bone marrow, and lung) are involved.

The outcome for children with LCH involving low-risk organs (skin, bones, lymph node, and pituitary gland) has always been excellent.

## 2. Case Report

A 6-year-old boy presented with a four-week history of right neck swelling. On clinical examination a 3 × 2 cm right cervical enlarged lymph node was noted. The patient did not have any other lymphadenopathy. There was no significant clinical history including weight loss or fever. Skeletal survey was normal. There were no osseous lytic lesions.

PET scan was reported to be normal.

Fine needle aspiration (FNA) of the right neck lymph node was performed. Cytology smears were prepared.

## 3. Materials and Methods

Diff-Quik (Medical Chemical Corp., Torrance, CA, USA) and Papanicolaou stains (Cardinal Health, Ontario, Canada) were performed. The residual pellet, after Cytospin processing, was fixed in 10% buffered neutral formalin for cell block preparation followed by hematoxylin and eosin (H&E) staining and ancillary studies.

Immunocytochemistry stains were performed on unstained sections of formalin fixed, paraffin embedded cell block by the standard avidin-biotin technique. The panel of antibodies used included CD1a, CD68, and S 100. 

## 4. Results

**Figure 1:**
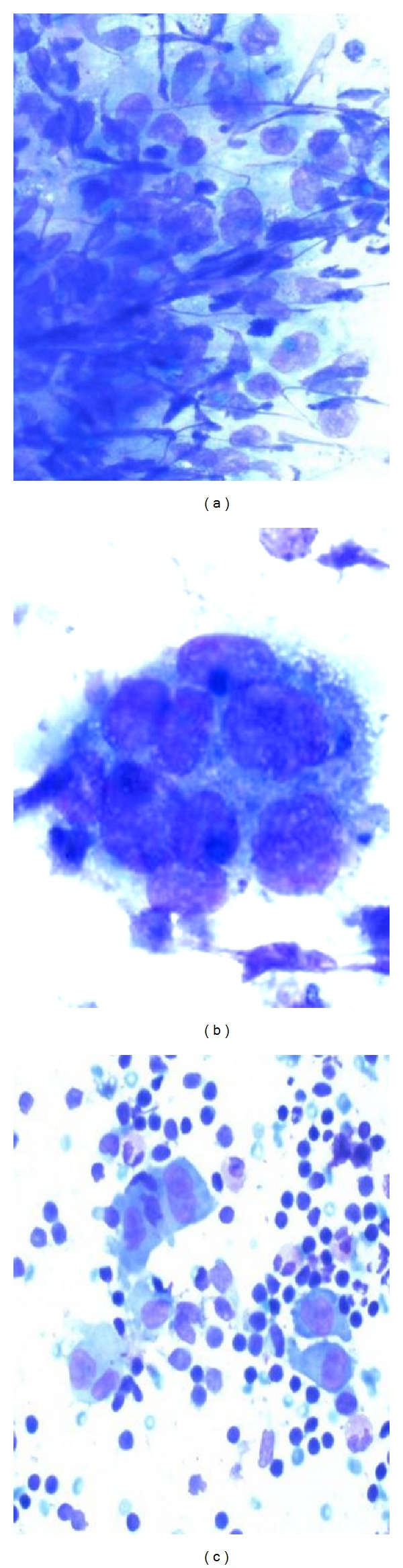
DQ-stained smears (×100, left), (×400 middle), and, (×100 right).

**Figure 2:**
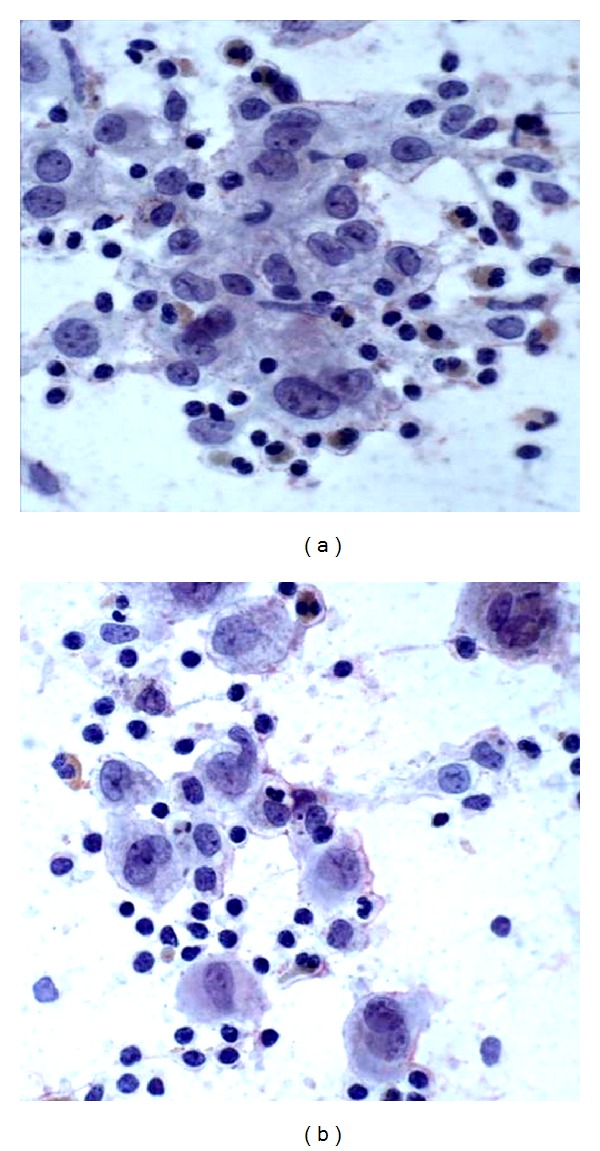
Pap-stained smear, ×200 (left), ×400 (right).

**Figure 3:**
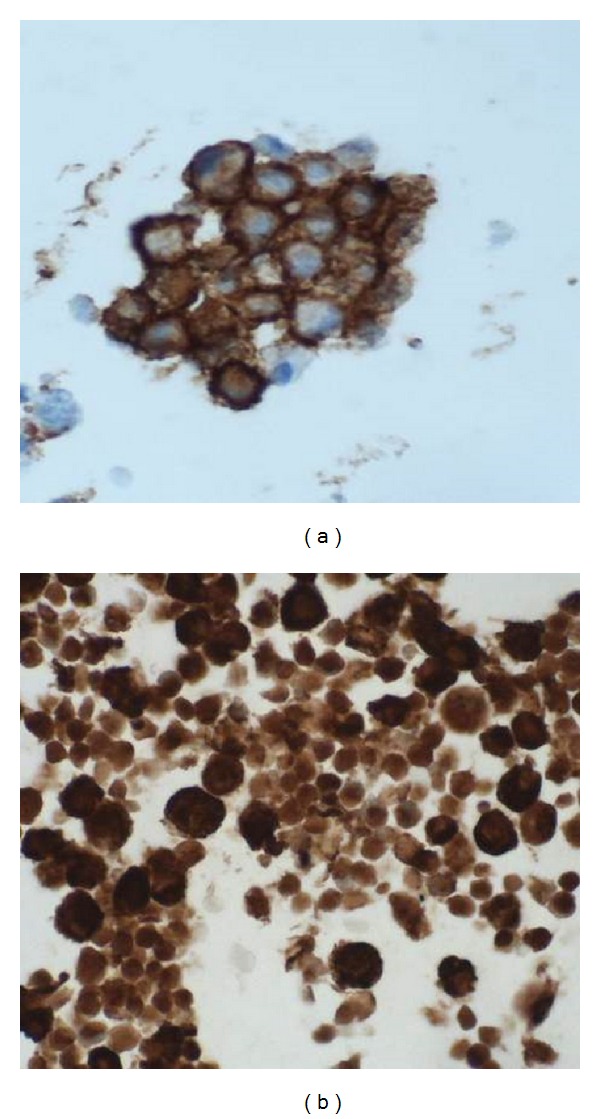
Ancillary studies of CD1a, S-100.

The cellular aspirate smears showed numerous single lying mononucleated and multinucleated histiocytes with abundant vacuolated cytoplasm and grooved, folded, and indented nuclei. The background consisted of abundant eosinophils, mixed lymphoid population, and lymphohistiocytic aggregates. No significant mitotic activity was noted. In Papanicolaou stain (Figure 2), the chromatin was fine and even, nucleoli were inconspicuous, and the cytoplasm was abundant and pale staining. In Diff-Quik (Figure 1), the Langerhan's cells had a plasmacytoid appearance, with eccentrically placed nuclei and abundant basophilic cytoplasm. Immunocytochemical stains were performed on the cell block and the tumor cells stained positively with CD1a, S-100 (Figure 3), and CD68. This immunoprofile was most consistent with Langerhan's cell histiocytosis.

## 5. Discussion

Langerhan's cell histiocytosis (LCH), or histiocytosis X, is a group of diseases characterized by proliferation of the Langerhan's cell. These disorders can involve many organ systems but primarily affect the bone, skin, lymph nodes, lungs, liver and spleen, endocrine glands, and nervous system. The separation of these conditions into separate entities, Letterer-Siwe disease, Hand-Schuller-Christian disease, and eosinophilic granuloma, is of historical interest [6]. The disease affects young children from 1 to 4 years but can present from birth to the ninth decade [6]. In a large series of 124 patients, bone, lymph node, and skin lesions were the most frequently seen, but 50% of patients showed liver disease and 23% lung disease with frequent hematological disease [7]. LCH involving lymph nodes usually occurs in patients in the pediatric age group with known systemic disease. However, rarely, LCH can primarily involve lymph nodes without other sites of the disease. 

In a series of 20 patients with Langerhan's cell histiocytosis involving lymph nodes, only 2 patients had restricted involvement of lymph nodes as in our case and all other cases were of lymph nodes from children with multisystemic disease [8].

The diagnosis of LCH in our patient was made on the basis of FNA smears of the lymph nodes. However, the differential diagnoses included conditions with localized aggregates of Langerhan's cells such as those observed in association with dermatopathic lymphadenitis (DL), parasitic infection, Kimura's disease, hypersensitivity reactions, cat-scratch disease, sinus histiocytosis with massive lymphadenopathy (SHML), and hyperplasic lymph nodes. In addition, on rare occasions, LCH can associate with a variety of malignant neoplasms in the same node, that is, lymphoma or metastatic neoplasms [9].

Langerhan's cells show positivity for S-100 and CD1a. Our case showed positivity for CD1a, S-100, and CD68. For localized lesions like lymph nodes, a simple, minimally invasive procedure with a low rate of complication is desirable. In view of this and the possibility of spontaneous resolution in localized disease, FNA alone could be used to confirm the diagnosis, in the correct clinical setting.

On one-year clinical followup, our patient did not have any other lymphadenopathy or bone involvement.

To conclude, the present case highlights the role of FNA in the diagnosis of the rare disease of LCH in a child. The cytologic features of LCH are highly characteristic to confirm the diagnosis with the help of immunocytochemistry. This can obviate the need of open biopsy and electron microscopy.

## References

[1] Laman JD, leenen PJ, Annels NE (2003). Langerhan’s -cell histiocytosis ‘insight into DC biology’. *Trends in Immunology*.

[2] McClain K, Jin H, Gresik V, Favara B (1994). Langerhans cell histiocytosis: lack of a viral etiology. *American Journal of Hematology*.

[3] Jeziorski E, Senechal B, Molina TJ (2008). Herpes-virus infection in patients with Langerhans cell histiocytosis: a case-controlled sero-epidemiological study, and in situ analysis. *PLoS ONE*.

[4] Salotti JA, Nanduri V, Pearce MS, Parker L, Lynn R, Windebank KP (2009). Incidence and clinical features of Langerhans cell histiocytosis in the UK and Ireland. *Archives of Disease in Childhood*.

[5] (1996). A multicenter retrospective survey of Langerhan’s cell histiocytosis: 348 cases observed between 1983 and 1993. The French Langerhan’s cell histiocytosis study group. *Archives of Disease in Childhood*.

[6] Odom RB, James WD, Berger TG (2000). *Andrews' Disease of the Skin*.

[7] Rivera-Luna R, Martinez-Guerra G, Altamirano-Alvarez E (1988). Langerhans cell histiocytosis: clinical experience with 124 patients. *Pediatric Dermatology*.

[8] Morgenfeld MC, Schajowicz F (1971). Solitary eosinophilic granuloma of lyumph node: five-year follow-up. *Pediatrics*.

[9] Neumann MP, Frizzera G (1986). The coexistence of Langerhans’ cell granulomatosis and malignant lymphoma may take different forms: report of seven cases with a review of the literature. *Human Pathology*.

